# Supplementary material for: Carbon limitation in hypereutrophic, periphytic algal wastewater treatment systems
Source: PLoS One. 2020 Oct 12;15(10):e0240525. doi: 10.1371/journal.pone.0240525 (PMC7549797; doi:10.1371/journal.pone.0240525)
Supplement: Attachment — Submitted filename: Manuscript.docx [file pone.0240525.s001.docx]

Manuscript-PONE-D-20-18899

Major comments

-I found ms is ordinary and not properly organized. Needs to provide more data in the form of figures and tables.

-No statistical figures are given in figures, also no legends provided.

-Information about algal species composition not given.
