# Supplementary material for: Carbon limitation in hypereutrophic, periphytic algal wastewater treatment systems
Source: PLoS One. 2020 Oct 12;15(10):e0240525. doi: 10.1371/journal.pone.0240525 (PMC7549797; doi:10.1371/journal.pone.0240525)
Supplement: Attachment — Submitted filename: MS-PONE.docx [file pone.0240525.s002.docx]

MS-PONE-D-20-18899R1

Reviewer is satified with the revised parts but has one major problem relating to stats used:

1. Why different (p) values used in the figures to define the level of significance. Normally p<0.05 used in published ms. I suggest to use similar level of significance for all figures.
