# Supplementary material for: Carbon limitation in hypereutrophic, periphytic algal wastewater treatment systems
Source: PLoS One. 2020 Oct 12;15(10):e0240525. doi: 10.1371/journal.pone.0240525 (PMC7549797; doi:10.1371/journal.pone.0240525)
Supplement: Attachment — Submitted filename: Keller_3ndCoverletter_PLOSOne_9-26-2020.docx [file pone.0240525.s003.docx]

9-26-2020

Re: Manuscript: PONE-D-20-18899R1

Dear Dr Leonidas Matsakas

Please accept this revised manuscript for consideration for publication in PLOS One. In order to accommodate comments from the reviewers, I have made all of the changes requested.

Specifically, I changed all of the figure descriptions to indicate that the p-values were p < 0.05. Originally, we reported the highest p-value of all of the pairwise comparisons (e.g., p <0.013 for all). Since these p-values varied among figures it created some confusion about the results. The edits we made should eliminate any confusion. These changes were tracked in the document. No other changes were requested or made to the document.

We hope these changes will make the manuscript acceptable for publication in PLOS ONE.

Respectfully submitted,

Troy A. Keller, https://orcid.org/0000-0003-0648-781X
